# Ophthalmology

**Published:** 1894-01-20

**Authors:** 


### OPHTHALMOLOGY.

*Antiseptic.*—The value of cyanide of mercury as an antiseptic was very warmly advocated by Schlösser at the Ophthalmological Society of Heidelberg.[1] He sums up its advantages as follows: It is much less irritating locally than perchloride. It very slightly, if at all, coagulates albumen. It is quite as powerful a germicide. Last, but not least, it does not spoil instruments. He recommends its use in a 2 per cent. solution in acute conjunctivitis and in chronic purulent lacryocystitis. In the former the lids are everted and touched with the solution, in the latter irrigation is employed.

Valude[2] has experimented on the use of formic aldehyde as an antiseptic. Like cyanide of mercury, it does not coagulate albumen, but it has the further advantage of being non-poisonous. Its aseptic properties are very great, and its sterilizing effects are said to last much longer than is the case with perchloride of mercury. He advocates its use as an antiseptic in operation cases in a watery solution of 1 to 2,000, and considers it of greater value than the perchloride in assisting post-operative infections and in the treatment of ophthalmia neonatorum. The sterilization of collyria is rendered safe and prolonged when the formic aldehyde is added in the proportion of 1 to 2,000. It forms an excellent bath for cleansing instruments, as it has no action on metals, and it has very slight, if any, irritating effect on the eye tissues.

*Cataract.*—The vexed question of cataract extraction, with or without iridectomy, seems no nearer a solution; some ophthalmologists who formerly were strongly in favour of iridectomy are now against it; whilst, on the other hand, some of the strongest advocates of "simple extraction" have returned to the other method. Mr. Teale,[3] in this year's Bowman Lecture, strongly favours operation by means of a shallow corneal flap without any iridectomy; but it is rather a significant fact that out of 89 cases so operated on, he subsequently performed iridectomy in 11. Only 58 were free from a secondary operation, either by iridectomy or discission. One very important point mentioned in his lecture was the value of puncture with a broad needle through cornea and iris into the vitreous, so as to allow a few drops to escape, as a means of checking irido-cyclitis after the needling of posterior capsular cataract.

Bell Taylor,[4] describing his operation, prefers section at the corneo-sclerotic junction as less likely to favour prolapse of the iris, and is strongly against iridectomy.

Galezowski[5] gives statistics of 1,934 cases, of which 1,599 were performed without iridectomy, and states that where the simple extraction is employed he obtains normal vision with lenses of 7 to 8 droptres, whereas, with iridectomy, 9 to 11 droptres are required. He considers that there is also less risk of prolapse of vitreous. In a paper written very shortly before,[6] he says: "The operation for cataract without iridectomy must be reserved for hard, total, or partial cataracts which have no complications, either of the individual or in the condition of the internal membranes of the eye," a very significant statement.

Higgins,[7] in a most impartial paper, giving results of both methods in 110 cases, says that, as a comparison, they are worthless, because "the whole of the non-iridectomy cases are picked ones." On all hands it seems to be the rule that where there is the slightest difficulty or danger apprehended, it is safer to do iridectomy; if that is so, should not in all cases added safety outweigh a possible and doubtful cosmetic advantage?

*Conjunctivitis.*—A peculiar form of conjunctivitis, following rapidly on the introduction of calomel into the conjunctival sac, forms the subject of a leading article in the *Therapeutic Gazette*[8], where cases are quoted reported by Fredenwald and Crawford[9]. It apparently is only induced where the patient is at the same time, or has been very lately undergoing treatment by iodide of potassium, and is presumably brought on by the combination of the eliminated drug in the lachrymal secretions with the calomel, and the production of a soluble salt of extremely irritating nature. The symptoms observed are swelling and hyperæmia of conjunctiva, profuse lachrymation, and in extreme cases there may be an actual sloughing of the membrane. The symptoms appear, as a rule, within an hour of the insufflation of the calomel, and the conjunctivitis is confined to the parts actually touched by the drug, usually the lower retrotarsal fold. The explanation offered by Fredenwald and Crawford is that calomel in the presence of animal fluids containing sodium chloride, aided by the body temperature, is converted into the bichloride and free mercury, and suggest that the action of calomel on phlyctenulæ is due to its activity in this nascent state. The bichloride so formed is then supposed to combine with the potassium iodide eliminated in the lachrymal secretion to form mercuric iodide, and so a double iodide of potassium and mercury is present, which is soluble in solutions of potassium iodide or sodium chloride, and which has a caustic action. Seeing how frequently calomel is used, it is well to bear in mind the possible danger in cases where the patient is or has recently been under the iodide treatment.

*Corneal Ulcers.*—Wreker[10] strongly advocates the treatment of corneal ulcers and abscesses by curetting and irrigation. He uses a small curette with sharp edges, and aims at removal of all sloughing material from the base and margins of the ulcer. He claims for this method that it at once alleviates pain and photophobia, that the cure is more rapid than by the use of antiseptics or actual cautery, and that a more transparent tissue results.

*Ophthalmia.*—The safest method of treating purulent ophthalmia is of manifest interest and importance to all practitioners, and during the past year much has been written on this subject. The general consensus of opinion has been overpoweringly in favour of the application of nitrate of silver, but the method of applying it and the strength best employed are points on which there is much disagreement.

Bettman[11] says that in the early stage, before any purulent secretion appears, it is unwise to use it, but in the later stages he advises application of a four grain to the ounce solution, and in severer cases up to forty and even sixty grains to one ounce. He uses it only once in twenty-four hours. He prefers a saturated solution of boracic acid for cleansing purposes, and employs cold pads where no corneal complications exist. As a preventive of ophthalmia neonatorum, vaginal douches of perchloride of mercury, or of weak carbolic acid, or Crede's method of a drop of 2 per cent. nitrate of silver instilled in the eyes of the new-born child. Brudenell Carter[12] has pointed out the dangers in using perchloride of mercury in these cases. He says that used in any strength sufficient to have germicidal properties it probably causes more mischief, and advocates the use of alum solution.

Hinde[13] characterises silver nitrate as "of all local remedies the most potent, and at the same time the least irritating," but in his opinion nothing stronger than a 2 per cent. solution is necessary; for irrigation purposes he is in favour of sublimate solution, 1 to 5,000.

Foucher[14] employs nitrate of silver fifteen grains to the ounce, irrigation with saturated boracic acid solution, or cyanide of mercury 1 to 1,500, but prefers the former. He is a strong advocate of the use of iced compresses, especially in ophthalmia neonatorum, even where corneal complications exist, but considers it unsafe where there is much chemosis.

Montgomery[15] uses very strong nitrate of silver, "at least sixty grains to the ounce," and if that does not lessen the purulent secretion, up to 120 grains. He condemns the use of syringe or pipette, but uses iced compresses for at least an hour after the application of the caustic. He prefers boracic acid, as being the least irritating and most efficacious for cleansing purposes.

*Optic Atrophy.*—The danger of optic atrophy following on facial erysipelas has been forcibly brought to notice during the past year by cases quoted by Snell and Scougal[16] at the Ophthalmological Society. In Mr. Snell's first case the left eye was more especially involved, and that eye only was lost. In his second case, double optic neuritis followed erysipelas with resulting atrophy. In Dr. Scougal's case the erysipelas was one-sided, but the neuritis and atrophy double.

Iodoform has been looked upon as such a safe antiseptic, that we feel inclined to wait for more evidence before condemning it as being the cause of the grave condition reported by Valude.[17] A child of twelve years sustained a severe burn of the greater part of the right side, and was dressed from the beginning with iodoform. Seven or eight months later the child suffered from diarrhœa, headache and vomiting, and loss of sight, leading to absolute blindness. Iodoform was stopped and salol substituted, when the other symptoms all subsided, but considerable amblyopia was present eleven months after the first symptoms of loss of sight. The condition was one of white atrophy of the optic nerves.

*Pannus.*—Two new methods of treating corneal pannus have been advanced lately, the first by Vignes, of Paris.[18] He first anæsthetises the cornea with cocaine, and then applies a thin layer of antipyrine by means of a brush or insufflator. This is followed by a rather sharp reaction, accompanied by lachrymation; after this has subsided gentle massage is applied through the closed lids. The process is said to be accompanied by no risk except where there is ulcerative or phlyctenular keratitis present, when it is contra-indicated, and he ascribes its effect on the pannus as being due to the hæmostatic properties of the antipyrine, which acting on the bloodvessels of the cornea cause their obliteration. Lydstone[19] employs powdered papoid with boric acid. Papoid, which is a digestive ferment, is said to have a digestive action on all proteid and albuminous substances, and that when applied to the pannus corneæ it brings about such a change in the tissues as to restore their transparency.

*Iritis.*—Quint has employed creasote administered internally in doses of five to twelve grains daily in cases of tubercular iritis with marked success.[20] It has usually been considered that where this disease has been diagnosed the risk of tubercular meningitis is so great that it is advisable to enucleate the eye, or in the case of an isolated tubercle to excise the portion of iris affected. When any doubt of the exact nature exists it may prove a very valuable method of treatment. Chauvel[21] considers that in syphilitic iritis mercury is best employed locally in the form of inunction.

*Retinitis.*—Bull contributes a very interesting paper to the *New York Medical Record* [22] giving the results of his observations of one hundred cases of *gouty retinal disorders.* His conclusions summarized briefly are: Firstly, that the fundal changes are invariably bilateral but rarely symmetrical. That as a rule gouty retinitis or neuro-retinitis never ends in blindness, the peripheral vision being seldom affected; the central vision undergoes marked impairment from the degeneration of the vessel walls. That the central loss of vision is progressive up to a certain point unless recognized and treated very early, and that after the disease is once established improvement cannot be looked for. Retinal hæmorrhages are rare except in the early stage. The principal fundal changes are due to sclerosis of the vessels of retina choroid and of the optic nerve. A well marked symptom is the yellowish granular exudation round the posterior pole, but which does not attack the macula itself. Finally he states that the degenerative changes in the optic nerve fibres are intra-ocular and cannot be traced any distance back.

*Retina, Detached.*—Detachment of the retina is usually regarded as a very hopeless condition to deal with. This is probably because it is so seldom seen until well established. A. and F. Boucheron,[23] writing on "The Radical Cure of Detachment of Retina," state that if quite recent *i.e.*, within six days, it can be treated with a fair prospect of success; but that after any greater lapse of time the prognosis is proportionately more unfavourable. According to them the most favourable cases for operative treatment are those in young subjects, where there is increased intra-ocular tension, when the upper portion is involved and only when the macula lutea is not involved. They advocate corneal section, sclero-corneal incision, or repeated paracentesis, with or without iridectomy, the object being to keep up a condition of prolonged low tension. At the same time employing constitutional treatment in syphilic or gouty cases. They do not advocate prolonged rest on the back.

[1] The Medical Week, Aug, 18, 1893. [2] Revue Générale D'Ophthalmologie, July, 1893. [3] Lancet, Juue 17, 1893. [4] Lancet, July 1, 1893. [5] Recueil d'Ophthalmologie, May, 1893. [6] Recueil d'Ophthalmologie, Dec., 1892. [7] Lancet, Nov. 11, 1803. [8] Therapeutic Gazette, Nov. 15, 1893. [9] American Journal of Ophthalmology, Aug. 1893. [10] Annales d'Oculistique, July, 1893. [11] The Clinic. Jour., Oct. 11, 1893. [12] Lancet, Oct. 29, 1892. [13] Medical Record, July 15, 1893. [14] Annals of Ophth. and Otology, Jan., 1893. [15] Ibid. [16] Medical Week, May 19, 1893. [17] Annales d'Oculistique, May, 1893. [18] Medical Week, March 17, 1893. [19] The Medical Standard, July, 1893. [20] Centralblatt f. pratt. Augenheilkunde, March, 1893. [21] Recueil d' Ophthalmologie, Jan., 1893. [22] N.Y. Medical Record, Aug. 5, 1893 [23] Archives d'Ophthalmologic, Feb., 1893.

